# Bibliographical

**Published:** 1891-11

**Authors:** 


### Bibliographical.

A Short Manual of Analytical Chemistry, Qualitative and Quantitative—Inorganic and Organic. Arranged on the principle of the course of instruction given at the South London School of Pharmacy, by John Muter, M.A., Ph.D., F.R.S.E., F.I.C., F.C.S. First American from the Fourth English Edition, edited by Claude C. Hamilton, M.D., Ph.G. Philadelphia; published by P. Blackiston, Son & Co., 1012 Walnut street, 1891.

This work is a very valuable one to the Analytical Chemist. This edition has been but slightly changed by the Reviser, except so far as seemed to him important, or even compulsory, by the

United States Pharmacopœia, except the chapter on Urine Analysis, which has been enlarged and illustrated.

The following are some of the subjects embraced in the table of contents, viz:

PART 1.—QUALITATIVE ANALYSIS.

Chapter 1.—The Processes Employed by Practical Chemists.

" 2.—Detection of the Metals.

" 3.—Detection and Separation of Acidulous Radicals.

" 4.—Qualitative Analysis, as applied to the Detection of Unknown Salts.

Chapter 5.—Qualitative Detection of Alkaloids, Glucosides, and certain Organic Bodies used in Medicine, with a General Sketch of Toxicological Procedure.

PART 2.—QUANTITIVE ANALYSIS.

Chapter 6.—Weighing, Measuring and Specific Gravity.

" 7.—Volumetric Quantitative Analysis and use of the "Nitrometer."

Chapter 8.—Gravimetric Quantitative Analysis of Metals and Acids.

Chapter 9.—Ultimate Organic Analysis.

" 10.—Special Processes for the Analysis of Water, Air and Food.

Chapter 11.—Special Processes for the Analysis of Drugs, Urine and Urinary Calculi.

Chapter 12.—Analysis of Gases, Polarisation and Spectrum Analysis.

The above will give an idea of the scope of the work. There are a large number of illustrations of Apparatus, for the various analytical processes, all of which are well described. The work is so arranged, and the subjects so well presented as to be a great aid in the work of the Analytical Chemist; every one engaged in this department of work would be aided by consulting this work, and especially those who are being initiated in the work of this science. It is well written, and presented in an attractive form.

